# ABT199/venetoclax synergism with thiotepa enhances the cytotoxicity of fludarabine, cladribine and busulfan in AML cells

**DOI:** 10.18632/oncotarget.28563

**Published:** 2024-03-14

**Authors:** Benigno C. Valdez, Bin Yuan, David Murray, Jeremy L. Ramdial, Uday Popat, Yago Nieto, Borje S. Andersson

**Affiliations:** ^1^Department of Stem Cell Transplantation and Cellular Therapy, The University of Texas MD Anderson Cancer Center, Houston, TX 77030, USA; ^2^Department of Oncology, University of Alberta, Edmonton, Alberta T6G 1Z2, Canada

**Keywords:** acute myeloid leukemia, pre-transplant regimens, venetoclax, thiotepa, busulfan

## Abstract

ABT199/venetoclax, an inhibitor of the pro-survival BCL-2 protein, has improved AML treatment. Its efficacy in hematopoietic stem cell transplantation (HSCT), when combined with other chemotherapeutic drugs, has not been thoroughly investigated. The present study demonstrates the synergistic cytotoxicity of ABT199/venetoclax with the DNA alkylator thiotepa (Thio) in AML cells. Cleavage of Caspase 3, PARP1 and HSP90, as well as increased Annexin V positivity, suggest potent activation of apoptosis by this two-drug combination; increased levels of γ-H2AX, P-CHK1 (S317), P-CHK2 (S19) and P-SMC1 (S957) indicate an enhanced DNA damage response. Likewise, the increased level of P-SAPK/JNK (T183/Y185) and decreased P-PI3Kp85 (Y458) suggest enhanced activation of stress signaling pathways. These molecular readouts were synergistically enhanced when ABT199/venetoclax and Thio were combined with fludarabine, cladribine and busulfan. The five-drug combination decreased the levels of BCL-2, BCL-xL and MCL-1, suggesting its potential clinical relevance in overcoming ABT199/venetoclax resistance. Moreover, this combination is active against P53-negative and FLT3-ITD-positive cell lines. Enhanced activation of apoptosis was observed in leukemia patient-derived cell samples exposed to the five-drug combination, suggesting a clinical relevance. The results provide a rationale for clinical trials using these two- and five-drug combinations as part of a conditioning regimen for AML patients undergoing HSCT.

## INTRODUCTION

Hematopoietic stem cell transplantation (HSCT) is a potentially curative treatment for hematological disorders [1]. Its success partly depends on the antitumor activity of the drugs used in the pre-transplant conditioning regimen. There is an urgent need to improve the efficacy of the pre-transplant regimen without jeopardizing patient safety. This can be accomplished by judiciously including (a) novel agent(s) with (a) different mechanism(s) of action. One such candidate drug is ABT199/venetoclax, a BH3-mimetic small molecule that binds to and inhibits the anti-apoptotic B-cell lymphoma 2 (BCL-2) protein, preferentially causing malignant cells to undergo apoptosis [2]. Preclinical studies have demonstrated the cytotoxicity of ABT199/venetoclax in leukemia cells [3]. ABT199/venetoclax is FDA-approved in combination with azacitidine or decitabine for the treatment of AML patients ≥75 years of age [4, 5].

Thiotepa (Thio) is an organophosphorus DNA alkylating agent used in the management of malignant solid tumors and hematologic malignancies, and particularly in HSCT [6]. The FDA designated Thio as a conditioning treatment prior to HSCT in 2007. Thiotepa has been successfully combined with other DNA alkylating agents and nucleoside analogs as conditioning regimens for HSCT [7–9]. Its potentially synergistic interaction with ABT199/venetoclax has not been previously investigated.

Busulfan (Bu) is another DNA alkylating agent used for conditioning therapy for HSCT [10]. Its combination with the nucleoside analogs fludarabine (Flu) and clofarabine (Clo) provides synergistic cytotoxicity as shown in preclinical and clinical studies [11–15]. Substitution of Clo with cladribine (Clad) is similarly cytotoxic in AML cells when combined with Flu and Bu *in vitro* [16]. When activated, Flu and Clad become incorporated into newly replicating DNA strands and inhibit DNA synthesis and repair, resulting in DNA strand break formation and histone modifications as well as chromatin remodeling. The associated unfolding of the chromatin structure potentially confers a greater susceptibility of the genomic DNA to Bu-mediated DNA alkylation and cross-linking [11], and attempts to repair DNA cross-links may also result in additional DNA strand breaks, such that the cycle which we have termed the “loop of death” model is further amplified by additional DNA alkylations, resulting in cell death [17]. The cytotoxicity of combined alkylating agent and nucleoside analog in AML cells is further enhanced when combined with ABT199/venetoclax, as previously reported [18].

On this basis, we hypothesized that combining Thio with the pro-survival inhibitor ABT199/venetoclax would effectively inhibit AML cell proliferation. We also hypothesized that further combining (Thio+ABT199) with the three cytotoxic chemotherapy drugs (Flu+Clad+Bu) would further increase their antileukemic efficacy. In addition to the ability of Thio to form DNA adducts, its radiomimetic action, which is believed to occur through the release of ethylenimine radicals and breakage of DNA bonds [19], has the potential to exacerbate the combined DNA injury. Cells that survive the cytotoxic effects of (Thio+Flu+Clad+Bu) may therefore not escape the effects of ABT199/venetoclax by inhibiting the pro-survival BCL-2 protein.

The present study reports the synergistic interactions of ABT199/venetoclax with Thio in the context of the treatment of AML. Moreover, the combination of ABT199/venetoclax and Thio enhances the cytotoxicity of (Flu+Clad+Bu) in AML cell lines and leukemia patient-derived cell samples. The results may provide relevant information for the design of clinical trials using these drugs to circumvent recognized drug-resistance mechanisms when used as part of pre-transplant conditioning regimens for AML patients undergoing allogenic HSCT.

## RESULTS

### Combination of thiotepa with ABT199/venetoclax exerts synergistic cytotoxicity towards AML cell lines

**Figure 1:**
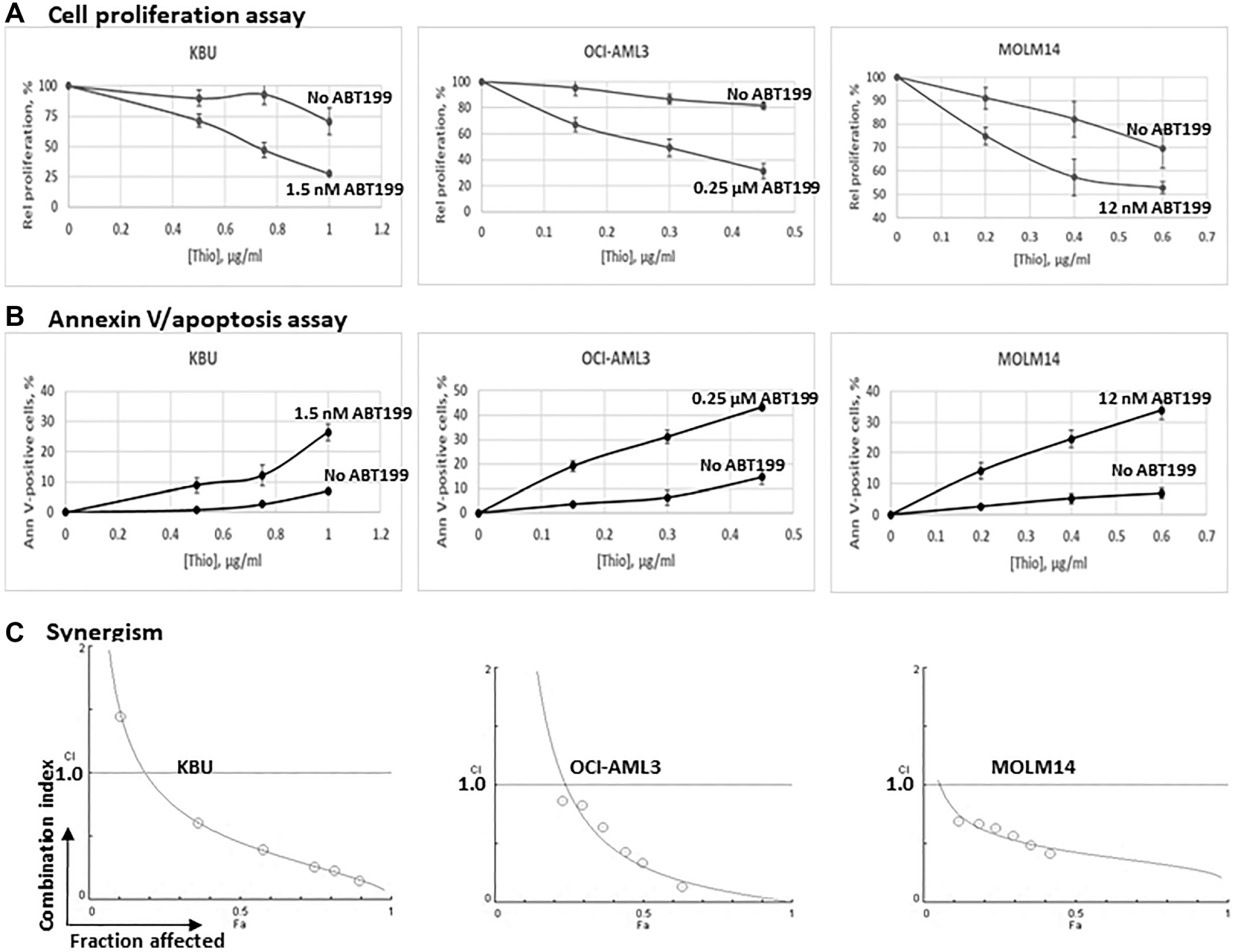
Synergistic cytotoxicity of thiotepa (Thio) and ABT199/venetoclax (ABT199) in AML cell lines. Cells were exposed to the indicated concentrations of Thio and ABT199 for 48 h and analyzed for (**A**) cell proliferation and (**B**) apoptosis by MTT and Annexin V assays, respectively. The results were normalized relative to untreated control cells for the Thio-only data and to ABT199-treated control cells for the (Thio+ABT199) data. (**C**) The synergistic cytotoxicity of Thio and ABT199 was quantitatively analyzed by exposing cells to various concentrations of the drugs, individually or in combination at constant ratio, for 48 h and cell proliferation was determined using the MTT assay. Combination indexes (CI) were determined as described under Materials and Methods. CI values less than 1 indicate synergism.

Exposure of the KBU, OCI-AML3 and MOLM14 cell lines to 1 μg/mL, 0.45 μg/mL, and 0.6 μg/mL Thio alone inhibited cell proliferation, relative to the control, by 29%, 18%, and 30%, respectively; addition of the indicated concentrations of ABT199/venetoclax further inhibited cell proliferation by 73%, 69%, and 47% relative to the control without Thio (Figure 1A). These results are consistent with the Annexin V assay. Exposure of KBU, OCI-AML3 and MOLM14 cell lines to 1 μg/mL, 0.45 μg/mL, and 0.6 μg/mL Thio (without ABT199) resulted in 7%, 15%, and 7% Annexin V positivity, and addition of ABT199/venetoclax increased the readings to 26%, 43%, and 34%, respectively (Figure 1B). These findings suggest that ABT199/venetoclax enhances the cytotoxicity of Thio in AML cell lines.

To test for possible synergistic interactions, the three AML cell lines were exposed to different concentrations of individual drugs, or to the drug combinations at a constant concentration ratio, and the cell proliferation assay was performed immediately after completion of the 48-h drug exposure. The combination index (CI) values at increasing drug effects were graphically analyzed according to the Chou-Talalay method as shown in Figure 1C. The calculated CI values for all 3 AML cells lines were generally less than 1, suggesting a significant synergism in these cell lines. At fraction affected (Fa) = 0.5 (50% inhibition of cell proliferation), the combination of Thio and ABT199/venetoclax resulted in CI values of 0.5. 0.3, and 0.4 in KBU, OCI-AML3 and MOLM14 cells, respectively; a further decrease in the CI values was observed at higher Fa values (Figure 1C), suggesting that a more meaningful synergism is achieved at higher drug concentrations and therefore may have clinical relevance.

### (Thio+ABT199/venetoclax) combination activates the apoptosis pathway and DNA damage response

**Figure 2:**
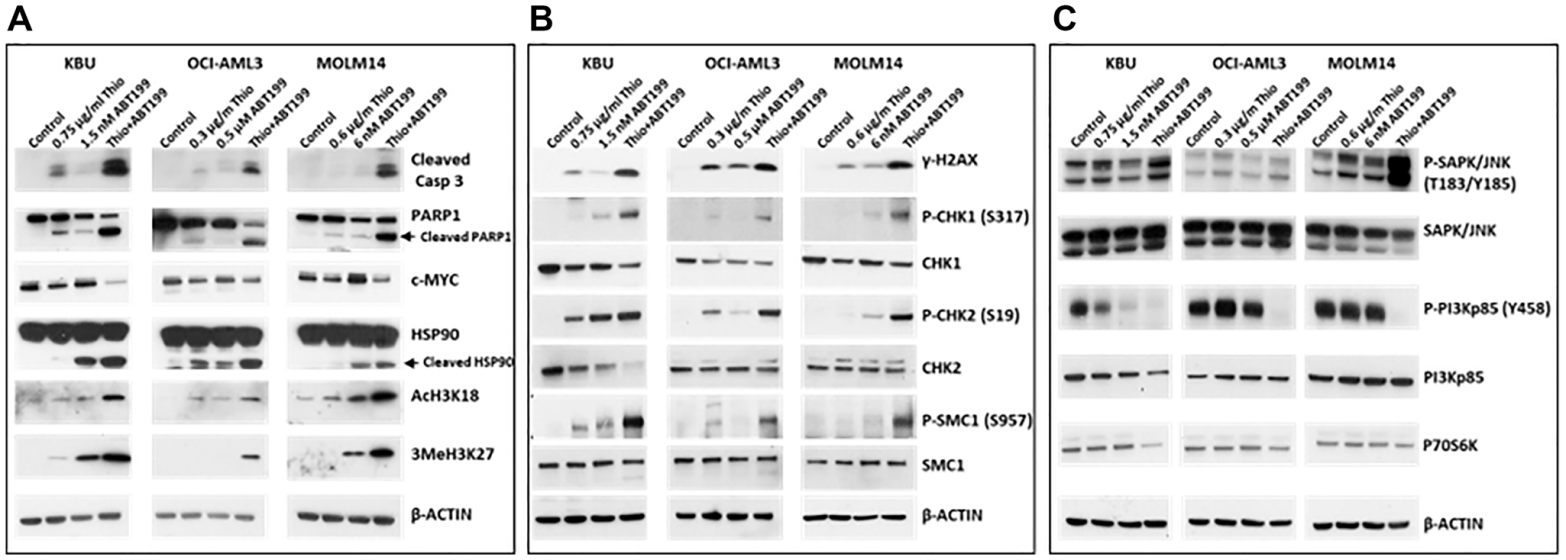
The combination of thiotepa (Thio) and ABT199/venetoclax (ABT199) activates (**A**) apoptosis, (**B**) DNA damage response, and (**C**) stress signaling pathways. Cells were exposed to drugs for 48 h and analyzed by Western blotting.

The observed increase in Annexin V positivity apparent in Figure 1B suggested activation of the apoptosis pathway in AML cells following the combined exposure to Thio and ABT199/venetoclax. We, therefore, assessed cell death in these cells by analyzing the cleavages of Caspase 3 and PARP1, known molecular markers of apoptosis [20]. A significant protein cleavage was observed in all three cell lines exposed to (Thio+ABT199/venetoclax) (Figure 2A). This activation of programmed cell death biomarkers correlated with a decrease in the level of pro-survival c-MYC and increased cleavage of heat-shock protein HSP90, a protein known to modulate apoptosis [21, 22]. The drug-mediated increase in the acetylation and methylation of histone 3 (AcH3K18 and 3MeH3K27) is indicative of chromatin remodeling (Figure 2A); such histone modifications may represent a histone code that directs the recruitment of proteins involved in the DNA damage response [23].

To determine the possible effects of (Thio+ABT199/venetoclax) on the DNA damage response, the phosphorylation of histone 2AX, a known molecular marker of DNA damage processing [24], was analyzed. Figure 2B shows an increased level of phosphorylated histone 2AX (γ-H2AX) in cells exposed to (Thio+ABT199/venetoclax). This finding is consistent with the observed increased phosphorylation of other known ATM substrates including CHK1, CHK2, and SMC1 (Figure 2B); ATM is one of the major apical kinases in cellular stress responses, notably in the processing of DNA double-strand breaks [25].

### (Thio+ABT199/venetoclax) combination activates stress-mediated signal transduction pathways

The observed activation of apoptosis and DNA damage response by the (Thio+ABT199/venetoclax) combination suggests the activation of stress-related pathways leading to cell death. We, therefore, sought to determine the effects of (Thio+ABT199/venetoclax) on the activation of the stress-activated protein/c-Jun N-terminal kinase (SAPK/JNK) pathway, which is known to transmit and convert stress signaling into apoptosis signaling in various cell types [26]. Increased phosphorylation of SAPK/JNK at threonine 183/tyrosine185, an indicator of its activation, was observed in KBU and MOLM14 cells, but not in OCI-AML3 cells, following exposure to (Thio+ABT199/venetoclax) (Figure 2C).

Activation of SAPK/JNK signaling has been reported to be inhibited by the PI3K/AKT pathway [27]. Intuitively, down-regulation of PI3K should therefore result in SAPK/JNK activation. Figure 2C shows that the drug-mediated increase in the phosphorylation of SAPK/JNK correlates with a decreased level of phosphorylated phosphoinositide 3-kinase regulatory subunit (P-PI3Kp85 (Y458)). Further, the activation of SAPK/JNK by phosphorylation is known to result in the phosphorylation of ribosomal protein S6 kinase beta-1 (P70S6K), which causes its destabilization and degradation [28], which is consistent with the observed modest decrease in the level of P70S6K after exposure to (Thio+ABT199/venetoclax) (Figure 2C).

### Effects of combining thiotepa and ABT199/venetoclax with other chemotherapy drugs in AML cells

**Figure 3:**
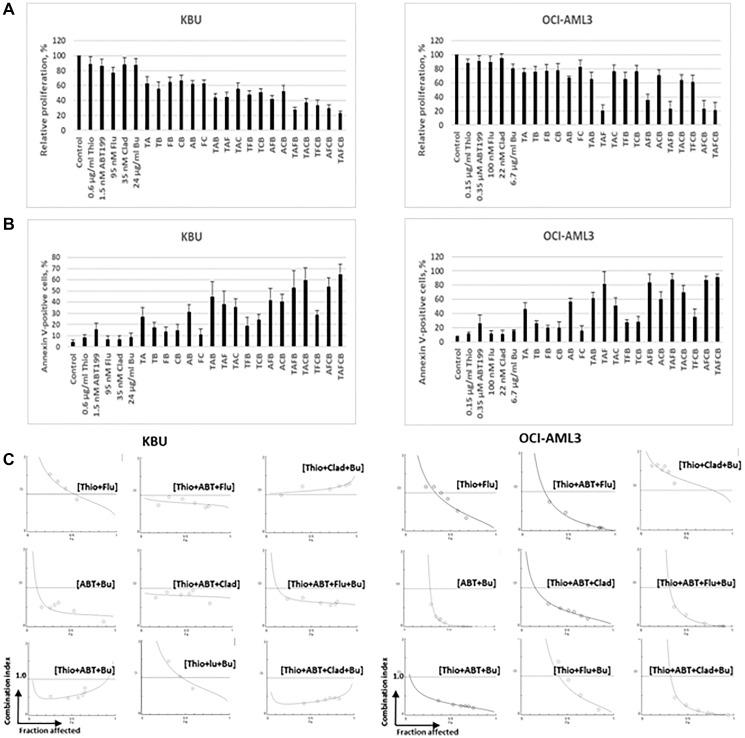
Addition of busulfan (Bu/B), fludarabine (Flu/F) and cladribine (Clad/C) increases the cytotoxicity of combined thiotepa (Thio/T) and ABT199/venetoclax (ABT199/A). Cells were exposed to the indicated drug concentrations for 48 h and analyzed for cell proliferation (Panel (**A**), MTT assay), and cell death (Panel (**B**), Annexin V assay). (**C**) Drug synergism was determined as described in Figure 1C.

Pre-clinical and subsequent clinical studies have shown the efficacy of combined Flu, Clo, and Bu in AML cells [11–15]. Cladribine has also shown similar cytotoxic activity to Clo in AML cells when combined with Flu and Bu [16]. It was therefore of great interest to determine if addition of (Flu+Clad+Bu) to (Thio+ABT199/venetoclax) would result in enhanced cytotoxicity towards AML cells. Using lower drug concentrations of Thio and ABT199/venetoclax (compared with those used in Figure 2), (Thio+ABT199/venetoclax) inhibited KBU and OCI-AML3 cell proliferation, relative to the control, by ~39% and ~23%, respectively; when combined with (Flu+Clad+Bu), the five-drug combination inhibited KBU and OCI-AML3 cell proliferation by ~78% and ~80%, respectively (Figure 3A). These results are consistent with the corresponding findings using the Annexin V apoptosis assay. Exposure of KBU and OCI-AML3 cells to (Thio+ABT199/venetoclax) increased Annexin V positivity by ~23% and ~39% compared with the control, respectively; exposure of KBU and OCI-AML3 cells to the five-drug combination increased Annexin V positivity by ~60% and ~84% relative to the control, respectively (Figure 3B). We then determined the combination indexes for Thio+ABT199 with Bu and individual nucleoside analogs; the CI values for (Thio+ABT199+Flu+Bu) and (Thio+ABT199+Clad+Bu) in KBU and OCI-AML3 cells were less than 1.0, suggesting synergistic cytotoxicity (Figure 3C).

### (Thio+ABT199/venetoclax+Flu+Clad+Bu+) combination activates apoptosis

**Figure 4:**
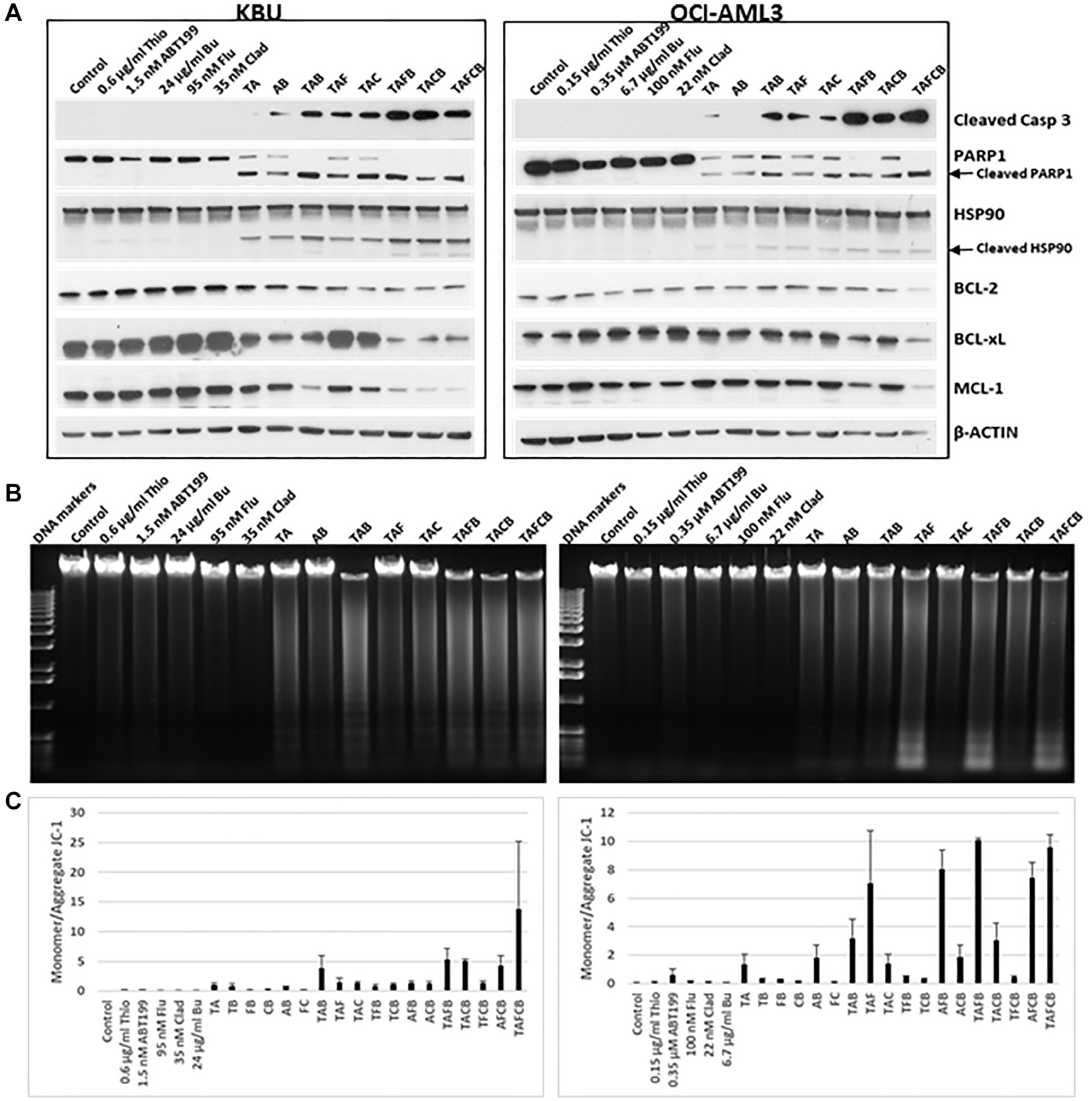
Effects of various drug combinations on the levels and modifications of proteins involved in cell death and survival. Cells were exposed to various drugs, individually or in combination, for 48 h and analyzed by (**A**) Western blotting, (**B**) agarose gel electrophoresis for DNA fragmentation, and (**C**) flow cytometry using JC-1 assay for changes in mitochondrial membrane potential. Abbreviations are the same as in Figure 3.

The increase in Annexin V-positive cells in the presence of the five-drug combination (Figure 3B) indicates marked induction of apoptosis in KBU and OCI-AML3 cells. This observation is further supported by the observed cleavage of PARP1, Caspase 3 and HSP90, and down-regulation of anti-apoptotic proteins, including BCL-2, BCL-xL and MCL-1 (Figure 4A). Moreover, exposure of cells to the five-drug combination increased DNA fragmentation, a biochemical hallmark of apoptosis [29], as determined by agarose gel analysis (Figure 4B), suggesting activation of caspase-dependent nuclear DNase.

To further determine the mechanism underlying drug-induced apoptosis, changes in the mitochondrial membrane potential (MMP) were analyzed. The relative monomeric (cytoplasmic) and aggregated (mitochondrial) forms of JC-1 were measured by flow cytometry: an increased monomeric/aggregated JC-1 ratio would suggest leakage of JC-1 from the mitochondria to the cytoplasm due to depolarization of the mitochondrial membrane. Exposure of KBU cells to (Thio+ABT199/venetoclax) and (Thio+ABT199/venetoclax+Flu+Clad+Bu) did indeed increase the ratio of monomeric/aggregated JC-1 from 0.042 (Control) to 1.0 and 13.8, respectively, suggesting a decreased MMP (Figure 4C). Exposure of OCI-AML3 cells to (Thio+ABT199/venetoclax) and (Thio+ABT199/venetoclax+Flu+Clad+Bu) likewise increased this ratio from 0.064 (Control) to 1.38 and 9.58, respectively; exposure of OCI-AML3 cells to (Thio+Flu+ABT199), (ABT199+Flu+Bu), (Thio+ABT199+Flu+Bu), and (ABT199+Flu+Clad+Bu) combinations resulted in a monomeric/aggregated JC-1 ratio similar to the five-drug combination (Figure 4C).

The observed decreases in MMP, activation of caspase 3, and chromosomal DNA fragmentation collectively suggest upregulation of pro-apoptotic factors that may contribute to the (Thio+ABT199/venetoclax+Flu+Clad+Bu)-mediated induction of apoptosis in AML cell lines.

### (Thio+ABT199/venetoclax+Flu+Clad+Bu) combination activates stress-mediated signal transduction pathways

**Figure 5:**
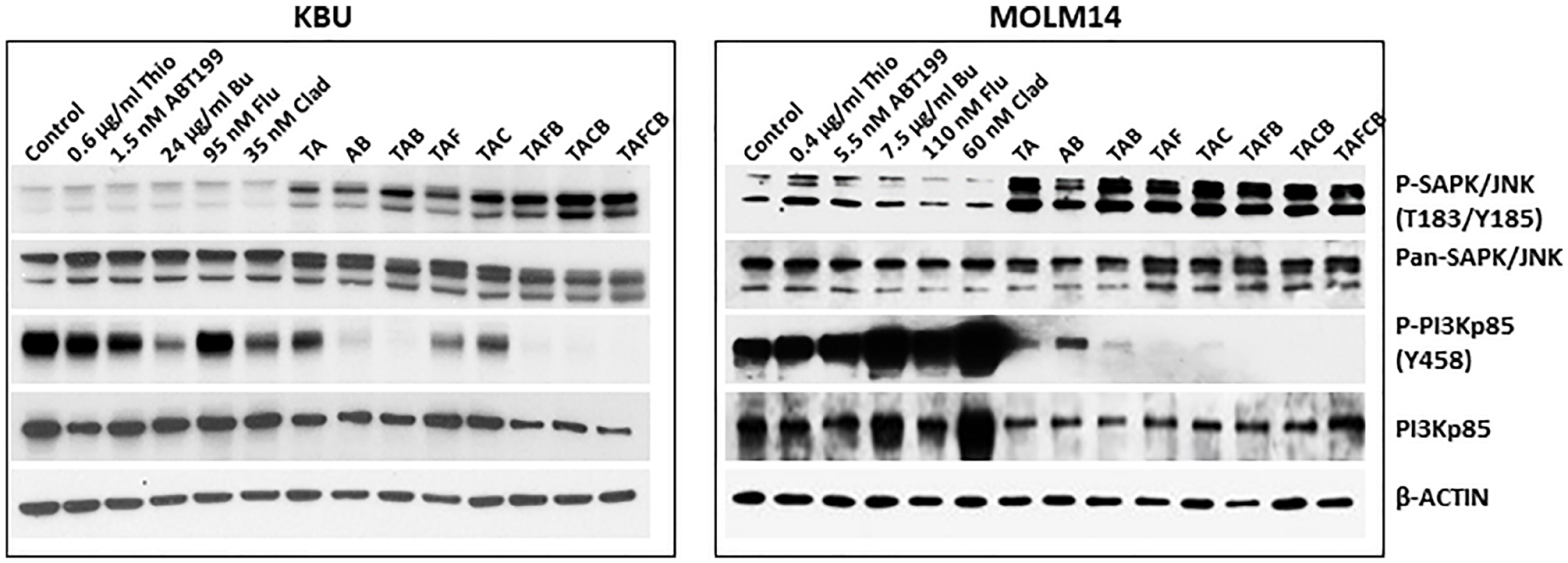
Effects of various drug combinations on the levels and modifications of proteins involved in key signal transduction pathways. Cells were exposed to various drugs, individually or in combination, for 48 h and analyzed by Western blotting. Abbreviations are the same as in Figure 3.

Since (Thio+ABT199/venetoclax) increased the phosphorylation of SAPK/JNK and decreased the level of P-PI3Kp85 (Y458) (Figure 2C), we sought to determine if the addition of (Flu+Clad+Bu) would enhance these events. Exposure of KBU cells to 0.6 μg/mL Thio and 1.5 nM ABT199/venetoclax (TA in Figure 5) increased the phosphorylation of SAPK/JNK and decreased the phosphorylation of PI3Kp85; these effects were further enhanced when cells were exposed to the five-drug combination (Figure 5), suggesting increased activation of stress-mediated signal transduction pathways. Similarly, exposure of MOLM14 cells to (Thio+ABT199/venetoclax) increased the phosphorylation of SAPK/JNK and decreased the level of P-PI3Kp85 (Y458), consistent with Figure 2C. Addition of (Flu+Clad+Bu) further decreased the level of P-PI3Kp85 (Y458) but did not enhance the phosphorylation of SAPK/JNK; all drug combinations decreased the level of PI3Kp85 (Figure 5).

### (Thio+ABT199/venetoclax+Flu+Clad+Bu) combination has similar synergistic effects in patient-derived leukemia cells

**Figure 6:**
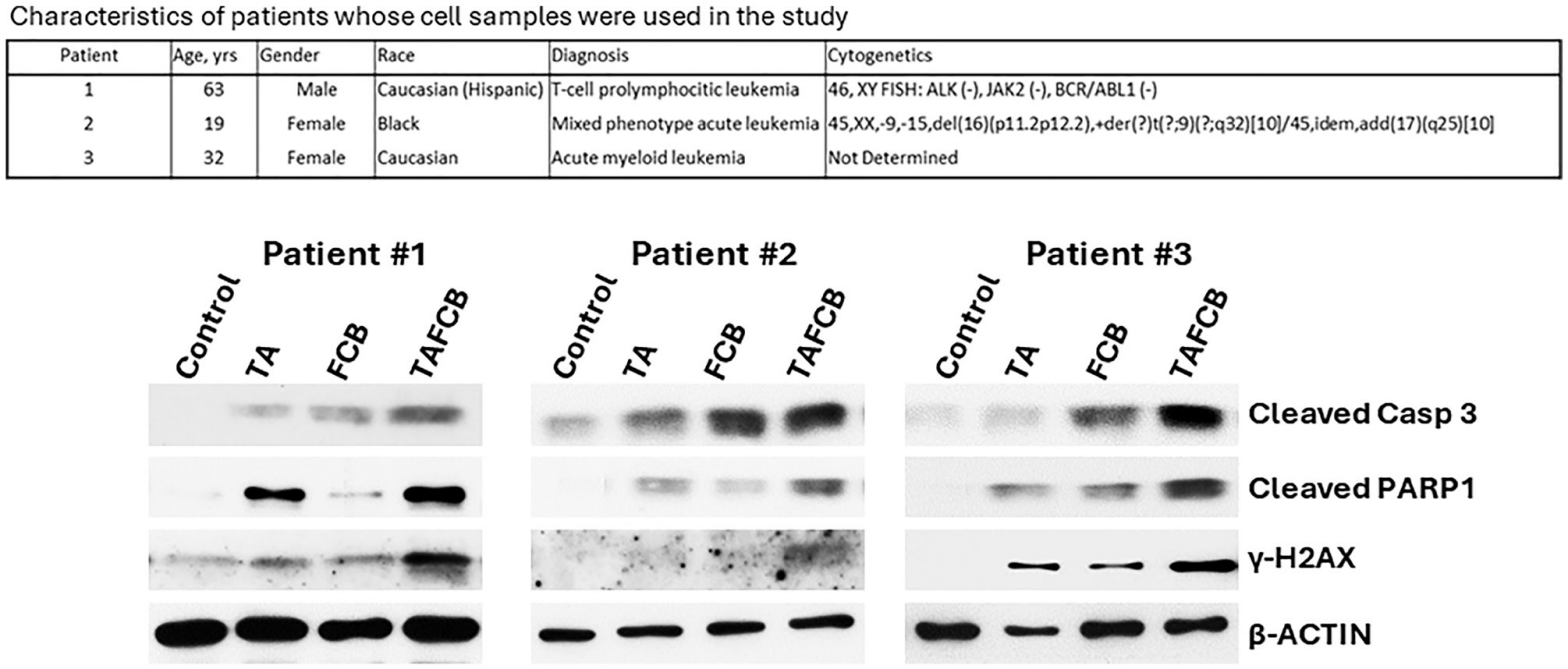
Effects of various drug combinations on molecular markers of apoptosis in patient-derived cell samples. Mononuclear cells were isolated from peripheral blood of patients with leukemia (upper panel) and exposed to the indicated drugs for 48 h prior to analysis by Western blotting (lower panel). Abbreviations are the same as in Figure 3. Drug concentrations: Patients 1 and 3: 0.8 μg/ml Thio, 50 nM ABT199, 15 μg/ml Bu, 0.18 μM Flu, 50 nM Clad; Patient 2: 0.6 μg/ml Thio, 20 nM ABT199, 12 μg/ml Bu, 0.18 μM Flu, 60 nM Clad.

To assess the potential clinical extension of our findings, cells from patients with leukemia were exposed to (Thio+ABT199/venetoclax), (Flu+Clad+Bu), or (Thio+ABT199/venetoclax+Flu+Clad+Bu) and analyzed by Western blotting. Increased cleavage of both Caspase 3 and PARP1 was observed in leukemia cells treated with the five-drug combination, suggesting enhanced activation of the apoptotic pathway (Figure 6). Phosphorylation of histone 2AX was also apparent in cells exposed to the five-drug combination, suggesting DNA double-strand break formation and activation of the DNA-damage response (Figure 6). These findings suggest synergistic effects of (Thio+ABT199/venetoclax+Flu+Clad+Bu) in cells derived from patients with leukemia involving mechanisms similar to those seen in the cultured cell lines.

## DISCUSSION

Preclinical and clinical studies have demonstrated the efficacy of combining ABT199/venetoclax with DNA hypomethylating agents and nucleoside analogs [30–34]. Our present study shows the synergistic interactions of ABT199/venetoclax with Thio in AML cell lines. The two-drug combination activates apoptosis, the DNA-damage response, and other signal transduction pathways related to cellular stress responses. These effects are further enhanced in combination with Flu, Clad and Bu.

The cytotoxicity of the (Thio+ABT199/venetoclax) combination is potentially mediated at the level of the initial DNA damage invoked by the alkylating agent Thio as suggested by the increased phosphorylation of histone 2AX, a known DNA damage molecular marker (Figure 2B) [24]. This observation is consistent with the increased phosphorylation of CHK1, CHK2 and SMC1, known substrates for ATM kinase (Figure 2B) [35]. The activation of the DNA-damage response (Figure 2B) is possibly involved in sending stress signals from the nucleus to the mitochondria leading to activation of Caspase 3 by cleavage (Figure 2A), concomitantly leading to activation of apoptosis. This finding is consistent with the activation of the stress-activated protein kinase/c-Jun N-terminal kinase (SAPK/JNK) signal transduction pathway (Figure 2C), which is known to transmit and convert stress signaling into apoptosis signaling in various cell types [26], and with the inhibition of phosphorylation of the PI3K regulatory subunit p85 (Figure 2C), a known cell survival protein which is constitutively activated by phosphorylation in most cancer cells [36]. Moreover, the Thio-mediated DNA damage likely resulted in the observed acetylation and methylation of histones (Figure 2A) and consequently induced chromatin remodeling, thereby enhancing the susceptibility of the genomic DNA to further alkylation by Thio.

All of these signal transduction events potentially lead to cell death unless they are overcome by the activity of pro-survival proteins such as BCL-2. Addition of a BCL-2 inhibitor such as ABT199/venetoclax thus presumably facilitated the commitment of the tumor cells to undergo apoptosis. The efficacy of such drug combinations is further shown by the cleavage of HSP90 and decreased level of c-MYC, two pro-survival proteins (Figure 2A) [37, 38].

The observed synergism of Thio and ABT199/venetoclax and the implied increased levels/complexity of DNA damage also provides a platform for combining these drugs with other chemotherapeutic agents. Our results show that combination of (Thio+ABT199/venetoclax) with (Flu+Clad+Bu) efficiently inhibited cell proliferation and strongly activated apoptosis in AML cell lines and patient-derived cell samples (Figures 3–6). We previously suggested that chromatin remodeling is an important intermediary in the efficacy and synergism of nucleoside analog-alkylating agent combinations [39]; in particular, nucleoside analogs (Flu and Clad) induce changes in the chromatin structure that confer a greater susceptibility of the DNA to alkylating agents (such as Thio and Bu). Although Thio and Bu can both potentially form DNA adducts and cross-linked DNA strands, the known radiomimetic activity of Thio [19] could additionally cause increased genomic insults by DNA strand cleavage. The combination of DNA damage and inhibition of the anti-apoptotic BCL-2 protein by ABT199/venetoclax likely contributed to committing the cells to apoptosis.

Our results have potential relevance in overcoming ABT199/venetoclax resistance in AML. Reprogrammed energy metabolism and overexpression of the antiapoptotic BCL-2 family members MCL-1 and BCL-xL have been partly associated with ABT199/venetoclax resistance [3, 40, 41]. Use of MCL-2 inhibitors may not be ideal for overcoming such resistance due to their associated cardiac toxicity [42, 43]. The ability of the (Thio+ABT199/venetoclax+Flu+Clad+Bu) combination to decrease the levels of BCL-2, BCL-xL and MCL-1 in both KBU and OCI-AML3 cells (Figure 4A) suggests that it might be useful as a combinatorial treatment for tumor cells that are resistant to ABT199/venetoclax.

Another challenge for practitioners in treating AML patients is the presence of inactive tumor suppressor P53 and positivity for FLT3-ITD mutations, both of which are associated with poor prognosis [44, 45]. Encouragingly, the synergistic interactions of (Thio+ABT199/venetoclax+Flu+Clad+Bu) were observed in P53-negative (KBU) and FLT3-positive (MOLM14) cell lines, which suggests that the five-drug combination might be efficacious in AML patients who are refractory to other treatments due to these mutations.

In conclusion, our results provide a molecular explanation for the synergistic cytotoxicity of ABT199/venetoclax and Thio, which may prove useful for induction therapy for AML. Moreover, addition of this two-drug combination to (Flu+Clad+Bu) may provide an even more efficacious pre-transplant regimen for AML patients undergoing HSCT.

## MATERIALS AND METHODS

### Cell lines and drugs

KBM3/Bu250^6^ (KBU) is an AML cell line established from one of our patients and made resistant to Bu as described previously [46]. The OCI-AML3 and MOLM14 AML cell lines were provided by Dr. Michael Andreeff’s laboratory (University of Texas MD Anderson Cancer Center (UTMDACC), Houston, TX, USA). Cells were grown in Roswell Park Memorial Institute (RPMI) 1640 medium (Thermo Fisher Scientific, Waltham, MA, USA) supplemented with 10% heat-inactivated fetal bovine serum (FBS: Gemini Bio-products, West Sacramento, CA, USA) and 100 IU/mL penicillin and 100 μg/mL streptomycin at 37°C in a humidified atmosphere of 5% CO_2_ in air. Thiotepa, ABT199/venetoclax, fludarabine and cladribine were purchased from Selleck Chemicals LLC (Houston, TX, USA). Busulfan was obtained from MilliporeSigma (St. Louis, MO, USA); it was dissolved in dimethylsulfoxide immediately prior to each experiment.

### Patient samples

Leukemia cell samples were isolated from patients’ peripheral blood using lymphocyte separation medium (Thermo Fisher Scientific) and incubated in suspension in the RPMI 1640 medium described above. Patient 1 had T-cell prolymphocytic leukemia (T-PLL), patient 2 had mixed phenotype acute leukemia and patient 3 had AML. Patient samples were collected after obtaining written informed consent, and all studies using these samples were performed under a protocol approved by the Institutional Review Board of the UT MD Anderson Cancer Center, in accordance with the Declaration of Helsinki.

### Cytotoxicity and apoptosis assays

Cells (6 mL of 0.5 × 10^6^ cells/mL) in T25 flasks were exposed to drugs, alone or in combination, for 48 h, aliquoted (100 μL) into 96-well plates and analyzed by the 3(4,5-dimethylthiazol-2-yl)-2,5-diphenyl tetrazolium bromide (MTT) assay [47]. Briefly, 30 μL of 2 mg/mL MTT reagent (MilliporeSigma) in phosphate-buffered saline (PBS) was added per well and incubated for 4 h at 37°C. The solid reaction product was dissolved by adding 100 μL of solubilization solution (0.1 N HCl in isopropanol containing 10% Triton X-100) to each well, mixing, and incubating at 37°C overnight. Absorbance at 570 nm was measured using a Victor X3 plate reader (Perkin Elmer Life and Analytical Sciences, Shelton, CT, USA). The number of metabolically active (MTT-positive) cells was determined relative to the control cells exposed to solvent alone.

Apoptosis was determined by flow-cytometric measurements of phosphatidylserine externalization [48] with Annexin-V-FLUOS (Roche Diagnostics, Indianapolis, IN, USA) and 7-aminoactinomycin D (BD Biosciences, San Jose, CA, USA) using a Muse Cell Analyzer (MilliporeSigma). Drug combination effects were estimated based on the combination index (CI) values [49] calculated using the CompuSyn software (Combo Syn, Inc., Paramus, NJ, USA). This program was developed based on the median-effect method in which a CI < 1 indicates synergy, a CI ≈ 1 is additive, and a CI > 1 suggests antagonism.

### Western blot analysis

Cells exposed to solvent or drug(s) were collected by centrifugation, washed with cold PBS, and lysed with cell lysis buffer (Cell Signaling Technology, Danvers, MA, USA). The protein concentrations were determined using a BCA Protein Assay kit (Thermo Fisher Scientific). Proteins were resolved on polyacrylamide-SDS gels and blotted onto nitrocellulose membranes (Bio-Rad, Hercules, CA, USA). Western blot analyses were done using the Immobilon Western Chemiluminescent HRP Substrate (MilliporeSigma). The sources of the antibodies and their optimum dilutions are available upon request.

### Analysis of changes in mitochondrial membrane potential (MMP)

Cellular changes in the MMP were assessed using a JC-1 (5,5′,6,6′-tetrachloro-1,1′,3,3′-tetraethylbenzimidazolylcarbo cyanine iodide) Mitochondrial Membrane Potential Assay Kit (Cayman Chemical, Ann Arbor, MI, USA). Cells were exposed to drug(s) for 48 h and 0.5 mL of cell suspension was aliquoted into 5-mL tubes. Diluted (1:10 with cell growth medium), 4 μL MMP-sensitive fluorescent dye JC-1 reagent was added to each tube, incubated at 37°C for 20 min, and immediately analyzed by flow cytometry (λ_ex_ = 488 nm) using the 530-nm (FL-1 channel, green) and 585-nm (FL-2 channel, red) band-pass filters simultaneously. Healthy cells with functional mitochondria and high MMP exhibit red fluorescence (aggregated JC-1), whereas cells with disrupted mitochondria and low MMP show green fluorescence (monomeric JC-1). The ratio of monomeric/aggregated JC-1 was then calculated.

## Abbreviations

AML: acute myeloid leukemia
Ann V: annexin V
BCL-2: B-cell lymphoma 2
Bu: busulfan
CI: combination index
Clad: cladribine
Clo: clofarabine
Fa: fraction affected
Flu: fludarabine
h: hours
γ-H2AX: phosphorylated histone 2AX
HSCT: hematopoietic stem cell transplantation
JC-1: 5,5′,6,6′-tetrachloro-1,1′,3,3′-tetraethylbenzimidazolyl carbocyanine iodide
min: minutes
MMP: mitochondrial membrane potential
MTT: 3-(4,5-dimethylthiazol-2-yl)-2,5-diphenyl tetrazolium bromide
SAPK/JNK: stress-activated protein kinase/c-Jun N-terminal kinase
Thio: thiotepa
